# Digital droplet PCR (ddPCR) for the precise quantification of human T-lymphotropic virus 1 proviral loads in peripheral blood and cerebrospinal fluid of HAM/TSP patients and identification of viral mutations

**DOI:** 10.1007/s13365-014-0249-3

**Published:** 2014-04-30

**Authors:** Giovanna S. Brunetto, Raya Massoud, Emily C. Leibovitch, Breanna Caruso, Kory Johnson, Joan Ohayon, Kaylan Fenton, Irene Cortese, Steven Jacobson

**Affiliations:** 1 Viral Immunology Section, Neuroimmunology Branch, National Institutes of Health, National Institute of Neurological Disorders and Stroke, 9000 Rockville Pike, Building 10, Room 5C-103, Bethesda, MD 20892 USA; 2 Institute for Biomedical Sciences, The School of Medicine & Health Sciences, The George Washington University, Ross Hall, 2300 Eye Street NW, Room 561, Washington, DC 20037 USA; 3 Bioinformatics Section, Information Technology & Bioinformatics Program, National Institutes of Health, National Institute of Neurological Disorders and Stroke, 9000 Rockville Pike, Building 10, Room 5S223, Bethesda, MD 20892 USA

**Keywords:** Human T-lymphotropic virus, Digital droplet PCR, Proviral load, HAM/TSP, Viral quantification

## Abstract

An elevated human T cell lymphotropic virus 1 (HTLV)-1 proviral load (PVL) is the main risk factor for developing HTLV-1-associated myelopathy/tropical spastic paraparesis (HAM/TSP) in HTLV-1 infected subjects, and a high cerebrospinal fluid (CSF) to peripheral blood mononuclear cell (PBMC) PVL ratio may be diagnostic of the condition. However, the standard method for quantification of HTLV-1 PVL—real-time PCR—has multiple limitations, including increased inter-assay variability in compartments with low cell numbers, such as CSF. Therefore, in this study, we evaluated a novel technique for HTVL-1 PVL quantification, digital droplet PCR (ddPCR). In ddPCR, PCR samples are partitioned into thousands of nanoliter-sized droplets, amplified on a thermocycler, and queried for fluorescent signal. Due to the high number of independent events (droplets), Poisson algorithms are used to determine absolute copy numbers independently of a standard curve, which enables highly precise quantitation. This assay has low intra-assay variability allowing for reliable PVL measurement in PBMC and CSF compartments of both asymptomatic carriers (AC) and HAM/TSP patients. It is also useful for HTLV-1-related clinical applications, such as longitudinal monitoring of PVL and identification of viral mutations within the region targeted by the primers and probe.

## Background

Human T cell lymphotropic virus 1 (HTLV-1), an oncogenic human retrovirus, is the causative agent of adult-T cell leukemia (ATL) and a myelopathy termed HTLV-1-associated myelopathy/tropical spastic paraparesis (HAM/TSP) (Gessain and Mahieux 2012). HTLV-1 has also been associated with other clinical manifestations including uveitis, myositis, and dermatitis (Gessain and Mahieux 2012). As with all other human retroviruses, HTLV-1 is transmitted from mother to child (mainly through breast milk), sexually, or from contact with infected blood (Kubota et al. 2000). It is currently estimated that 10 to 20 million people worldwide are infected with HTLV-1 (Gessain and Mahieux 2012). The majority of infected individuals will remain lifetime asymptomatic carriers (AC), while up to 5 % will develop either HAM/TSP or ATL (Proietti et al. 2005). Regions that are highly endemic for HTLV-1 infection include the Caribbean, Japan, and regions of South America and Africa (Proietti et al. 2005; Gessain and Mahieux 2012).

HTLV-1 predominantly infects T cells (CD4^+^ and CD8^+^) and establishes a reservoir in the memory/effector and regulatory CD4^+^ T cells (Araya et al. 2011; Demontis et al. 2013). Unlike other retroviruses, HTLV-1 replicates mainly through clonal expansion of the infected cells rather than through the retroviral life cycle with de novo cell-to-cell infection. An elevated HTLV-1 proviral load (PVL)—a high percentage of provirus-carrying cells in the peripheral circulation—is a major risk factor for developing HAM/TSP in HTLV-1-infected subjects (Furtado Mdos et al. 2012) and is also found in patients with ATL (Archanjo et al. 2011). The peripheral blood mononuclear cell (PBMC) PVL is 16-fold higher in HAM/TSP patients compared to AC, and the risk of developing HAM/TSP increases exponentially once the PBMC PVL exceeds 1 % (Nagai et al. 1998). Additionally, HAM/TSP patients are characterized by an even higher PVL in the cerebrospinal fluid (CSF) compared to the PBMC (Demontis et al. 2013). This high PVL is associated with multiple immunologic abnormalities that contribute to the development of HAM/TSP. When compared to AC, HAM/TSP patients have higher levels of spontaneous lymphocyte proliferation (Sakai et al. 2001; Pinto et al. 2011) increased HTLV-1 expression, and importantly, a higher frequency of HTLV-1 specific CD8^+^ T cells (Kubota et al. 2000; Yamano et al. 2002), which are thought to be critical mediators of central nervous system (CNS) injury.

Reliable quantification of HTLV-1 PVL in the PBMC, serum, CSF, and other tissues is critical for understanding the clinical associations with this agent and for monitoring viral infection and evaluating therapeutic interventions in HTLV-1-infected patients. Importantly, quantification of CSF PVL in the context of HTLV-1 infection and neurologic symptoms is of diagnostic utility. For example, HAM/TSP patients have an elevated CSF to PBMC PVL ratio compared to HTLV-1-infected subjects with other neurologic disorders, such as multiple sclerosis (MS) (Nagai et al. 2001b). Real-time PCR (referred to here as quantitative PCR or qPCR) is currently the standard method of PVL quantification (Hayden et al. 2013; Strain et al. 2013). However, this technique has inherent limitations that may preclude precise and accurate quantification of viral load (Hayden et al. 2013). Particularly, the dependence of quantification on extrapolation from a standard curve often results in high inter-assay variability and, at times, difficulty in accurately measuring the PVL in compartments with low numbers of cells, such as the CSF (Lee et al. 2004). An indirect approach of determining target DNA quantity via a standard curve is not optimal for viral load quantification, especially as consistency and reliability are essential for detecting biologically meaningful thresholds and changes.

A novel technique, digital droplet PCR (ddPCR), allows for the direct absolute quantification of a target gene in a given sample. A restriction enzyme is initially used to fragment the DNA in each sample. The PCR samples are partitioned into thousands of nanoliter-sized droplets and then subsequently amplified on a thermocycler. All droplets are then queried for fluorescent signal (Pinheiro et al. 2012). Each independent event (droplet) is defined as either positive or negative for the target probe by the amplitude of its recorded fluorescence signal (Hindson et al. 2011). Due to the random, independent segregation of DNA fragments into droplets, Poisson algorithms can be used to determine absolute copy numbers in the original sample independently of a standard curve (Pinheiro et al. 2012).

This direct approach to target gene quantification suggests that ddPCR may provide a more precise and reliable method for PVL quantification, particularly when applied to samples with low numbers of cells. The partitioning of each sample into thousands of droplets allows for many events to be queried for fluorescent signal in a single well, thereby increasing the overall precision of quantification per sample (Pinheiro et al. 2012). Several recent studies have found that ddPCR successfully quantifies the viral loads of CMV in plasma and HIV in PBMC (Hayden et al. 2013; Strain et al. 2013). In this study, we examined the reliability of ddPCR in quantifying HTLV-1 PVL in the PBMC and CSF cells of HTLV-1-infected individuals.

## Results

### Characterization of ddPCR for the detection of HTLV-1 *tax*

**Fig. 1:**
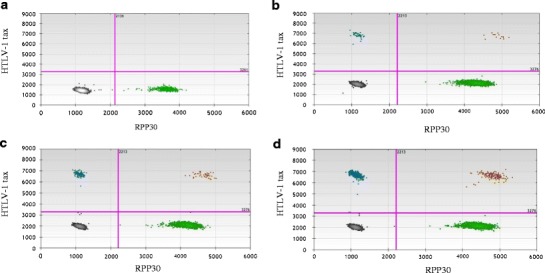
Representative two-dimensional ddPCR plots of individuals with increasing HTLV-PVL. **a** PBMC DNA from a normal blood donor. **b** PBMC DNA from an HTLV-1-infected individual with a PVL of 3.25 %. **c** PBMC DNA from an HTLV-1-infected individual with a PVL of 8.46 %. **d** PBMC DNA from an HTLV-1-infected individual with a PVL of 19.50 %

Figure 1 displays four representative two-dimensional ddPCR plots from individuals with varying HTLV-1 positivity in PBMC DNA. The *x*-axis displays the amplitude of VIC fluorescence, corresponding to the housekeeping gene *RPP30*, while the *y*-axis displays the amplitude of FAM fluorescence, corresponding to HTLV-1 *tax*. For each plot, droplets in the lower left quadrant are negative for both targets (black), droplets in the upper left quadrant are positive for HTLV-1 *tax* only (blue), droplets in the lower right quadrant are positive for *RPP30* only (green), and droplets in the upper right quadrant are positive for both HTLV-1 *tax* and *RPP30* (brown).

Figure 1a displays the result from representative normal healthy donor PBMC DNA, containing only the housekeeping gene without amplifiable HTLV-1 *tax*. Figure 1b–d shows the plots from PBMC DNA of three representative HAM/TSP patients with increasing HTLV-1 PVL that range from 3.25 to 19.5 %.

### Characterization of ddPCR for HTLV-1 PVL quantification in PBMC

**Fig. 2:**
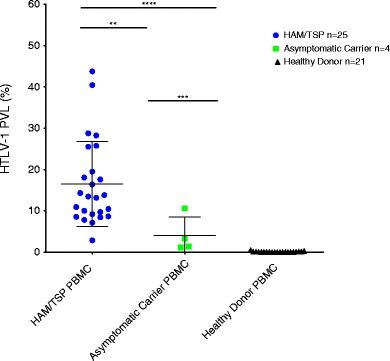
Statistically significant differences in HTLV-1 PBMC PVL between HAM/TSP patients, asymptomatic carriers, and healthy donors. Mann-Whitney tests were performed to compare differences between groups in sets of pairs. HAM/TSP PBMC PVL were statistically different from asymptomatic carrier PBMC PVL, *p* = 0.0093 (**). HAM/TSP PBMC PVL were also statistically different from healthy donor PBMC PVL, *p* < 0.0001 (****). Asymptomatic carrier PBMC PVL versus healthy donor PBMC PVL were also statistically different, *p* = 0.002 (***)

Following the initial characterization of the assay as shown in Fig. 1, ddPCR HTLV-1 PVL quantification was extended to PBMC DNA from 25 HAM/TSP patients, four AC, and 21 healthy donors (Table 1), (Fig. 2). The PVL was calculated using the following formula:

$$ \mathrm{PVL}=\left(\frac{\mathrm{quality}\kern0.15em \mathrm{of}\kern0.15em \mathrm{HTVL}-1\kern0.5em \mathrm{tax}}{\left(\frac{\mathrm{quantity}\;\mathrm{of}\;\mathrm{housekeeping}\;\mathrm{gene}}{2}\right)}\right)\times 100. $$

The median PBMC PVL for HAM/TSP patients was 13.5 % (range 3–44 %) compared to 2.3 % for AC (range 1–11 %), which is consistent with previous reports of higher PVL in HTLV-1 infected individuals with neurologic disease compared to AC (Nagai et al. 1998). Although the cohort of AC is small (a known limitation to studying HTLV-1 in non-endemic areas), the median PBMC PVL was significantly different between HAM/TSP patients, AC, and healthy donors (Kruskal-Wallis test, *p* = 5.77 × 10^−09^; degrees of freedom = 2). When analyzed in pairs, the PBMC PVL differences between these patient populations and healthy donors were statistically significant (Mann-Whitney tests, HAM/TSP versus AC *p* = 0.0093; HAM/TSP versus healthy donors *p* < 0.0001; healthy donors versus AC *p* = 0.002).

### Lower inter-assay variability of ddPCR compared to qPCR

**Fig. 3:**
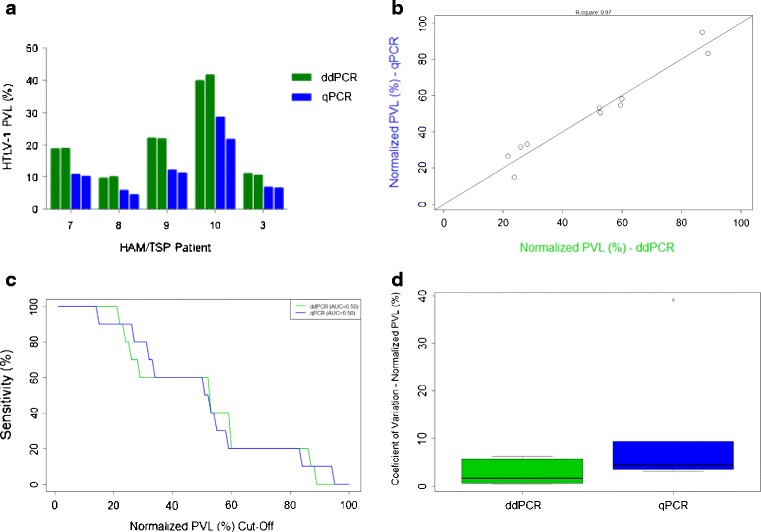
Strong correlation between ddPCR and qPCR for HTLV-1 PBMC PVL quantitation, though ddPCR has lower inter-assay variability. **a** Replicate, non-normalized PBMC PVL values obtained for five HAM/TSP patients using qPCR and ddPCR were similar, though the values obtained by ddPCR were consistently higher. **b** A strong correlation exists between the normalized PVL values for ddPCR and qPCR (Pearson correlation coefficient, *R* ^2^ = 0.97). **c** An ROC analysis of the normalized PVL values for each method indicates that the assays perform similarly for HTLV-1 PVL estimation. **d** Comparison of each method’s normalized values demonstrates that ddPCR has a lower coefficient of variation compared to qPCR

Though the goal of this study was to characterize a new assay for HTLV-1 PVL quantification, a comparison of ddPCR and quantitative real-time (qPCR) was performed, as qPCR is the most commonly used method of measuring HTLV-1 PVL. PBMC DNA from five HAM/TSP patients was run on two separate plates by both ddPCR and qPCR to directly compare the inter-assay variability of these two methods (Fig. 3a). The PVL of these individuals ranged from 9.4 to 39.8 % by ddPCR and from 5.0 to 28.4 % by qPCR. As the PVL measurements were higher by ddPCR (*p* = 0.0152, paired *t* test), the values were normalized such that the assays could be compared.

Post-normalization, the assays are highly correlated (Pearson correlation coefficient, *R* ^2^ = 0.97) (Fig. 3b), with similar performance by ROC analysis (Fig. 3c). Using the normalized PVL, we found a lower coefficient of variation for ddPCR compared to qPCR (Fig. 3d), suggesting stronger inter-assay reliability of ddPCR.

### Low intra-assay variability of ddPCR

**Fig. 4:**
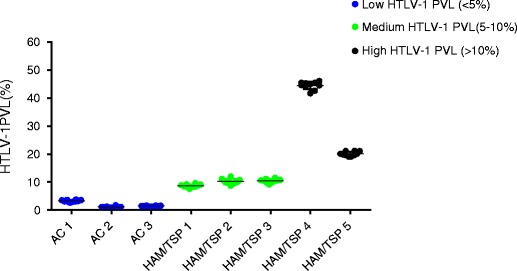
Low intra-assay variability by ddPCR over a wide range of HTLV-1 PBMC PVL. The intra-assay variability of ddPCR for low, medium, and high PVL groups was evaluated by running ten replicates of PBMC DNA from eight patients (three AC and five HAM/TSP). For the low PVL group (<5 %), the mean PVL was 1.85 % with a mean CV of 13.7 %. For the medium PVL group (5–10 %), the mean PVL was 9.77 % with a mean CV of 7.44 %. For the high PVL group (>10 %), the mean PVL was 32.245 with a mean CV of 3.4 %

To further characterize the precision and reliability of ddPCR for quantification over a wide range of HTLV-1 PVL, the intra-assay variability was evaluated. HTLV-1 PBMC PVL of eight infected individuals was measured by ddPCR in sets of ten replicates on one plate (Fig. 4). Samples were categorized into three subgroups, similar to previous reports (Demontis et al. 2013): low PVL (<5 %), medium PVL (5–10 %), and high PVL (>10 %) (representative plots of each subgroup are shown in Fig. 1b–d). Three individuals with PVL values less than 5 % were HTLV-1 positive AC, while the five individuals with medium and high PVL were HAM/TSP patients. The mean HTLV-1 PVL for individuals in the low, medium, and high groups were 1.95, 9.77, and 32.35 %, respectively, with mean coefficients of variation of 13.7, 7.44, and 3.4 %, respectively. These data suggest that low and acceptable CV can be achieved at all HTLV-1 PVL ranges, with the highest precision and reliability observed in the medium to high PVL ranges (>5 %), which includes the majority of HAM/TSP patients (Fig. 2).

### Elevated PVL in the CSF of HAM/TSP patients

**Fig. 5:**
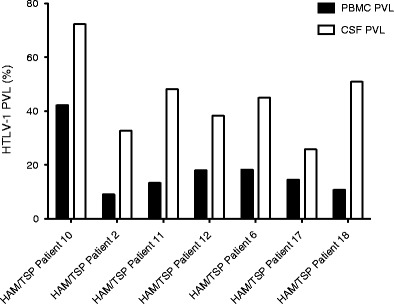
Elevated CSF to PBMC PVL in HAM/TSP patients demonstrated by ddPCR. HTLV-1 PVL is elevated in the CSF of seven HAM/TSP patients relative to matched PBMC, by approximately 2.8-fold. For each patient, there is a significant difference between PBMC and CSF HTLV-1 PVL (paired *t* test, *p* = 0.0036)

As HTLV-1 is cell-associated, persisting as an integrated provirus, HTLV-1 PVL quantification is challenging in compartments with low numbers of cells, such as CSF. Digital droplet PCR was used to quantify CSF PVL, which was then compared to matched PBMC from seven HAM/TSP patients. As shown in Fig. 5, CSF PVL was greater than matched PBMC for each patient (approximately 2.8-fold, see Table 2). The mean CSF PVL, 47.88 %, was significantly greater than the mean PBMC PVL, 20.71 % (paired *t* test, *p* = 0.0036). This finding is in agreement with previous reports of an elevated CSF PVL to PBMC PVL ratio in HAM/TSP patients (Nagai et al. 2001b; Demonti et al. 2013).

**Table 1:** Patient demographics

Subject	Sex	Age	Ethnicity	Disease duration	Disability score (EDSS^a^/IPEC^b^)
HAM-1	F	25	Afro-Caribbean	17 years	8/20
HAM-2	F	50	Caucasian	1 year	1.5/14
HAM-3	F	71	Caucasian	6 years	3/7
HAM-4	M	61	African-American	16 years	6/11
HAM-5	F	52	Afro-Caribbean	7 years	6.5/17
HAM-6	F	52	Afro-Caribbean	5 years	6/14
HAM-7	M	74	Afro-Caribbean	22 years	6/12
HAM-8	F	64	Caucasian	3 years	6.5/14
HAM-9	F	44	African-American	10 years	1.5/8
HAM-10	M	52	Afro-Caribbean	19 years	7/21
HAM-11	M	56	Afro-Caribbean	5 months	8/24
HAM-12	M	65	Caucasian	9 years	6/12
HAM-13	F	63	Afro-Caribbean	16 years	6/14
HAM-14	F	61	Afro-Caribbean	9 years	5/13
HAM-15	F	44	Afro-Caribbean	6 years	1.5/1
HAM-16	M	61	African	15 years	6.5/16
HAM-17	F	53	Afro-Caribbean	8 years	7/21
HAM-18	F	61	Hispanic	6 years	8/25
HAM-19	F	36	Afro-Caribbean	16 years	8/24
HAM-20	F	46	Afro-Caribbean	15 years	6/18
HAM-21	M	41	Caucasian	3 years	7.5/24
HAM-22	F	50	Afro-Caribbean	12 years	7/23
HAM-23	F	63	Afro-Caribbean	6 years	1.5/4
HAM-24	F	56	Afro-Caribbean	6 years	3.5/6
HAM-25	M	67	Caucasian	24 years	6.5/12
AC-1	F	65	Afro-Caribbean	N/A	N/A
AC-2	M	58	Hispanic	N/A	N/A
AC-3	F	41	Caucasian	N/A	N/A
AC-4	F	28	Afro-Caribbean	N/A	N/A

^a^ *EDSS* Expanded disability status scale

^b^ *IPEC* Instituto de Pesquisa Clinica Evandro Chagas disability scale

### Longitudinal monitoring of HTLV-1 PVL using ddPCR

**Fig. 6:**
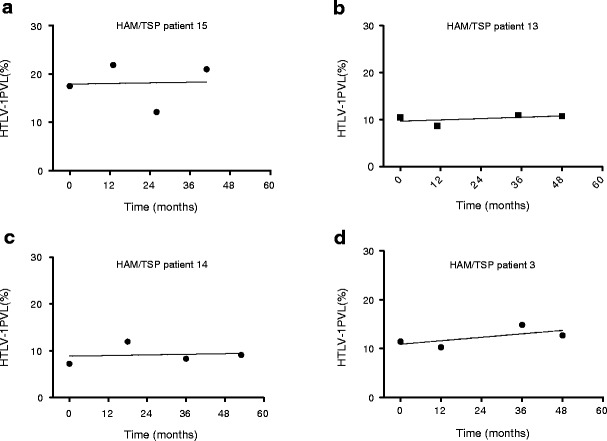
HAM/TSP patients’ PBMC PVL are relatively stable over a period of approximately 4 years. **a** HAM/TSP patient 15 had a mean PVL of 18.13 %. **b** HAM/TSP patient 13 had a mean PVL of 12.3 %. **c** HAM/TSP patient 14 had a mean PVL of 9.16 %. **d** HAM/TSP patient 3 had a mean PVL of 10.25 %

Given the precision and reliability of ddPCR for HTLV-1 PVL quantification, longitudinal PBMC samples from four HAM/TSP patients were tested to determine PVL variation over time. For each patient, PBMC samples collected approximately 1 year apart for 4 years were run in duplicate (Fig. 6). All patients were slow progressors with stable disability and HTLV-1 PVL in the medium to high range. Although in all HAM/TSP patients the HTLV-1 PVL remained relatively stable over this 4-year time period, we observed several CV greater than expected for the corresponding PVL (Fig. 4). For example, patient 14, with a mean PVL of 9.16 %, falls into the medium PVL range as defined in this paper (5–10 %), and is therefore expected to have a CV of 7.44 % (Fig. 4). However, in this longitudinal analysis, this patient was found to have a CV of 22.10 %. Similarly, patient 3 has a mean PVL of 12.3 % and is therefore categorized in the high PVL range, with an expected CV of 3.4 %. However, this patient was found to have a CV of 10.17 %.

### Detection of HTLV-1 sequence mutations using ddPCR

**Fig. 7:**
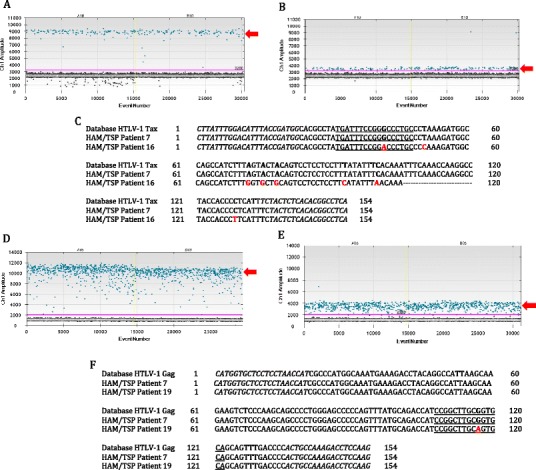
DdPCR fluorescence profile indicates mutations in viral region targeted by primers/probe. **a** A typical one-dimensional droplet profile for HAM/TSP patients has a positive HTLV-1 *tax* population at a fluorescence amplitude of 9,000 (*red arrow*). **b** The HTLV-1 *tax*-positive droplets for HAM/TSP patient 16 were observed around an amplitude of 3,500 (*red arrow*). **c** Sequence alignment revealed that HAM/TSP patient 16 has eight point mutations (indicated in red) in the region of HTLV-1 *tax* targeted by the ddPCR primers/probe, one in the probe-binding region. The primer-binding regions are italicized, and the probe-binding region is underlined. **d** A typical one-dimensional droplet profile for HAM/TSP patients has a positive HTLV-1 *gag* population at a fluorescence amplitude of 11,000 (*red arrow*). **e** The HTLV-1 *gag* positive droplets for HAM/TSP patient 19 were observed around an amplitude of 4,000 (*red arrow*). **f** Sequence alignment revealed that HAM/TSP patient 19 has one point mutation (indicated in *red*) in the region targeted by the *gag* probe (*underlined*)

In addition to serving as a highly reliable tool for quantifying HTLV-1 PVL, ddPCR was shown to be useful for detecting mutations in the target gene. As described previously, the fluorescence amplitude of the positive droplets reflects primer/probe binding and is specific to each primer/probe set. In this study, we observed that the fluorescence amplitude of *tax*-positive droplets in a singleplex reaction was approximately 9,000 for all HTLV-1 infected individuals tested (Fig. 7a, red arrow). However, for one HAM/TSP patient (patient 16), the amplitude of the *tax*-positive droplets was unusually low, between 3,500 and 4,000 (Fig. 7b, red arrow). When multiple assay runs and DNA extractions confirmed this unusual droplet profile, the HTLV-1 *tax* target sequence from this subject was analyzed. Eight point mutations (highlighted in red, Fig. 7c) were found within the region targeted by the ddPCR HTLV-1 *tax* primers/probe. One of these mutations was in the probe-binding region, suggesting that changes in positive fluorescence amplitude may indicate sequence mutations in this region. This application of ddPCR has allowed the identification of HTLV-1 mutations in other regions of the virus, such as the *gag* gene, as shown for patient #19 in Fig. 7d–f.

## Discussion

HTLV-1 PVL, or the frequency of infected cells in a given sample, is an important biomarker of disease in the context of HTLV-1 infection. A high PVL in the PBMC is a risk factor for developing HAM/TSP in HTLV-1 infected individuals, although there is significant PVL overlap between AC and HAM/TSP patients (Nagai et al. 1998; Yamano et al. 2002; Olindo et al. 2005; Waters et al. 2011; Demontis et al. 2013). A high PVL in patients with neurologic disease has been suggested to drive an activated immune response that may play a role in disease pathogenesis (Ijichi et al. 1993). Monitoring of PVL is therefore important, particularly during the evaluation of interventional therapies.

A commonly used method for PVL quantification is real-time quantitative PCR (qPCR). This technique extrapolates from standard curves to quantify viral target and housekeeping genes and is limited by sensitivity and high inter-assay variability, particularly at the lower range of target input. Due to these limitations, a third-generation digital droplet PCR technology that allows for direct HTLV-1 PVL quantification was developed. In this study, we compared the precision and reproducibility of ddPCR to qPCR, demonstrated the utility of ddPCR for the detection of HTLV-1 in samples with low cell numbers (such as CSF), and identified a use for ddPCR for the identification of viral mutations in HAM/TSP patient samples.

Digital droplet PCR was shown to be a highly precise and reproducible method of HTLV-1 PVL quantification. The coefficient of variation was low, less than 10 %, over a wide range of PVL. This favorably compares, if not exceeds, reliability of qPCR (Demontis et al. 2013; Grassi et al. 2013). Importantly, the coefficient of variation of ddPCR was lower than that of qPCR. Such assay characteristics are critical when quantifying HTLV-1 PVL and may have clinical consequence, particularly in patients with low PVL or low input of cellular DNA. While the present study was not designed to demonstrate superiority of any PCR methodology, direct comparison of normalized HTLV-1 PVL measurements by ddPCR and qPCR showed a strong correlation between the two methods, although ddPCR demonstrated higher HTLV-1 PVL than qPCR. This may reflect differences in PCR assay platforms, different regions of HTLV-1 and housekeeping target gene amplification, and primer/probe design requirements (Grassi et al. 2013).

The results of the present study are consistent with previous reports that HAM/TSP patients have significantly higher HTLV-1 PVL than AC or healthy controls in PBMC (Nagai et al. 1998; Olindo et al. 2005; Demontis et al. 2013), even though there were low numbers of AC in this report, a common limitation of studies performed in non-HTLV-1 endemic areas. Importantly, the measurement of HTLV-1 PVL in the CSF of infected individuals with neurologic symptoms has been shown to be diagnostically useful (Puccioni-Sohler et al. 2007). Elevated levels of HTLV-1 in the CSF compared to PBMC have been shown to differentiate HAM/TSP from patients with other neurologic disease and HTLV-1 infection (Puccioni-Sohler et al. 2007). However, the precise quantification of HTLV-1 PVL in compartments with a paucity of cells such as CSF is challenging when using PCR-based assays that measure the amount of cellular target based on a standard curve. By contrast, ddPCR uses Poisson algorithms to determine absolute copy numbers independently of a standard curve, which results in lower inter-assay variability (Pinheiro et al. 2012). In all seven HAM/TSP patients with matched CSF and PBMC samples, the CSF PVL was shown to be significantly higher than the PBMC PVL, with an average 2.8-fold increase of virus in the CSF. Moreover, in this small cohort of HAM/TSP patients, we observed that the three patients with earlier and more aggressive disease had the highest fold increase in the CSF/PBMC ratio (Fig. 5, patients 2, 11, 18; fold increase >3.5). The remaining four patients, with the lowest fold increase in the CSF/PBMC ratio had more stable, chronic disease (Table 2). If confirmed in larger cohorts, this observation could be of diagnostic and prognostic value.

**Table 2:** CSF/PBMC PVL ratios correlation with disease course

HAM/TSP patient	Symptom onset (years prior to CSF collection)	Neurologic disability		Disease course	Age at CSF collection	PBMC PVL (%)	CSF PVL (%)	CSF/PBMC PVL ratio	CSF white cell count
		IPEC^a^	EDSS^b^						
10	18	21	7	Slow progression	53	42.25	72.34	1.71	2
17	10	21	7	Initially rapid course then slow progression	54	14.61	25.84	1.77	6
12	9	12	6	Slow progression	65	18.08	38.28	2.12	16
6	6	13	6.5	Slow progression	54	18.35	45.06	2.46	2
11	1	24	8	Rapid course	57	13.46	48.21	3.58	52
2	2	13	1.5	Rapid course	50	9.06	32.7	3.61	9
18	1	12	6	Rapid course	38	10.8	50.95	4.72	14

^a^ *IPEC* Instituto de Pesquisa Clinica Evandro Chagas disability scale

^b^ *EDSS* Expanded disability status score

The precision and reliability of our ddPCR assay also allowed for the evaluation of longitudinal variation of PBMC PVL in HAM/TSP patients. In four HAM/TSP patients, we have shown that the PBMC PVL remained relatively stable over a period of approximately 4 years (Fig. 6). While minor fluctuations in PBMC HTLV-1 PVL were observed over time, the biological significance of these changes remains unclear, as they did not correlate with clinical or radiological findings. However, since ddPCR is a robust method with low intra- and inter-assay variability, it will be useful for long- and short-term monitoring of patient PVL during and after clinical intervention.

Though primarily utilized for PVL quantification, we recognized an additional use of ddPCR for the detection of mutations in the target gene sequence, as fluorescence amplitude reflects the binding affinity of the primers and probe to the target DNA. Two HAM/TSP patients were shown to have differences in the fluorescence amplitude (one for HTLV-1 *tax*, one for HTLV-1 *gag*) compared to 25 HAM/TSP patients with characteristic fluorescence amplitudes for both viral gene targets (Fig. 7). Sequence analysis of the HTLV-1 *tax* target region for HAM/TSP patient 16 demonstrated eight point mutations (one in the probe-binding region), while the HTLV-1 *gag* target region (Fig. 7c) for HAM/TSP patient 19 showed one mutation in the probe-binding region (Fig. 7f). While these viral mutations did not appear to correlate with any clinical or atypical presentation of HAM/TSP, this finding highlights a heretofore-unappreciated application of ddPCR for the detection of viral mutants.

## Conclusions

In conclusion, our study demonstrates that ddPCR is a precise, reliable method for HTLV-1 PVL quantification. This technique can be used to accurately quantify the PVL in PBMC and CSF samples of HTLV-1 infected individuals. Importantly, it can be utilized to study the relationship between PVL in a patient’s CSF and PBMC, monitor PVL fluctuations over time, and confidently assess the effects of various clinical interventional therapies on PVL. Interestingly, the fluorescence amplitude of a given target gene was shown to allow easy identification of viral mutations in patient samples. We are currently designing primers and probe sets specific for other regions of the HTLV-1 genome to allow for differentiation between HTLV-1 and HTLV-2 in a single multiplex reaction.

## Methods

### Patients

All samples used in this study were collected from subjects followed at the Neuroimmunology Branch of the National Institute of Neurologic Disorders and Stroke in Bethesda, MD under a protocol studying the natural history of HTLV-1 infection (protocol #98N0047). This study included 25 HAM/TSP patients, 4 HTLV-1AC, and 21 healthy volunteers (Table 1). Prior to study inclusion, written informed consent was obtained from each subject in accordance with the Declaration of Helsinki. The study was reviewed and approved by the National Institutes of Health institutional review board.

### Samples

PBMC were isolated from whole blood using Ficoll-Hypaque (Lonza, Walkersville, MD) centrifugation (1,200 rpm, 30 min, room temperature). Following isolation, 3 × 10^6^ cells were washed with PBS, pelleted, and frozen at −80 °C until use. To isolate CSF cells, 5–10 mL of CSF were centrifuged (1,300 rpm, 10 min, 4 °C) immediately after collection by lumbar puncture. The CSF supernatant was carefully removed, and the cell pellet was washed in PBS and stored at −80 °C until use. DNA was extracted from the PBMC and CSF cell pellets using DNeasy Blood and Tissue kit (Qiagen, Valencia, CA) according to the manufacturer’s instructions. NanoDrop 1000 (Thermo Scientific, Wilmington, DE) was used to measure the DNA concentration and purity. The extracted DNA was then used for both ddPCR and qPCR HTLV-1 PVL quantifications.

### Primer/probe design

HTLV-1 *tax* primers and FAM/MGB probe were designed for ddPCR using NCBI Primer Blast and Primer3Plus (Table 3). These primers amplify a 154-base-pair region of HTLV-1 *tax* (NCBI Gene ID 1491938). A temperature gradient experiment was performed to determine the optimal annealing temperature of 59 °C (data not shown). Additionally, in a duplex PCR, we amplified a cellular housekeeping gene, ribonuclease P protein subunit P30 (*RPP30*, NCBI Gene ID 10556) (Hindson et al.), which was detected with a VIC/MGB probe (Table 3). The final concentrations in the ddPCR reaction were 900 nM of each primer and 250 nM of each probe.

**Table 3:** Primer/probe sequences

PCR platform	Gene name	Primer sequences	Probe sequence
qPCR	HTLV-1 *tax*	Fwd: ACAAAGTTAACCATGCTTATTATCAGC Rvs: ACACGTAGACTGGGTATCCGAA	6FAM TTCCCAGGGTTTGGA CAGAGTCTTCT TAMRA
ddPCR	HTLV-1 *tax*	Fwd: CTTATTTGGACATTTACCGATG Rvs: TGAGGCCGTGTGAGAGTAGA	6FAM TGATTTCCGGGCCCTGC MGBNFQ
	HTLV-1 *gag*	Fwd: CATGGTGCTCCTCCTAACCA Rvs: CTTGGAGGTCTTTGGCAGTG	6FAM CCGGCTTGCGGTGCA MGBNFQ
	*RPP30*	Fwd: GATTTGGACCTGCGAGCG Rvs: GCGGCTGTCTCCACAAGT	VIC CTGACCTGAAGGCTCT MGBNFQ

For HTLV-1 PVL quantification by qPCR, a different set of HTLV-1 *tax* primers and FAM/TAMRA probe were used to amplify a 79base-pair region of the *tax* gene (Table 3) (Nagai et al. 2001a). The qPCR assay included a standard curve for HTLV-1 *tax* and a cellular housekeeping gene, *beta-actin*. The standard curve for HTLV-1 *tax* was derived from TARL-2 murine cell line DNA, known to carry the *tax* gene (pX) at one copy per cell (1 μg of TARL2 DNA contains 1.65 × 10^5^ copies of the pX gene) and included five points with a 1:10 serial dilution (40,000, 4,000, 400, 40, and 4 copies). For the beta-actin standard curve, we used β-actin Detection Reagents (Applied Biosystems, Foster City, CA), which included control human DNA and TaqMan primers and a FAM/TAMRA probe. The standard curve was derived from five points containing 30300, 3030, 303.3, 30.3, and 10 copies of *beta-actin*. The final concentrations in the qPCR reactions were 900 nM of each primer and 250 nM of the probe for HTLV-1 *tax*, and 100 nM of each primer and 66.6 nM of the probe for *beta-actin*.

### Digital droplet PCR (ddPCR)

DNA was digested with the restriction enzyme BamH1 (New England Biolabs, Ipswich, MA) for 30 min at 37 °C, diluted 1:5 with PCR-certified water. The digested diluted DNA was mixed with the HTLV-1 *tax* (or *gag*) and *RPP30* primers and probes and Bio-Rad 2× Supermix, which was then emulsified with droplet generator oil (Bio-Rad, Hercules, CA) using a QX-100 droplet generator according to the manufacturer’s instructions. The droplets were then transferred to a 96-well reaction plate (Eppendorf, Hauppauge, NY) and heat-sealed with pierceable sealing foil sheets (Thermo Fisher Scientific, West Palm Beach, FL). The duplex PCR amplification was performed in this sealed 96-well plate using a GeneAmp 9700 thermocycler (Applied Biosystems, Grand Island, NY) with the following cycling parameters: 10 min at 95 °C, 40 cycles consisting of a 30-s denaturation at 94 °C and a 60-s extension at 59 °C, followed by 10 min at 98 °C and a hold at 12 °C.

Following PCR amplification, the 96-well plate was transferred to a QX100 droplet reader (Bio-Rad, Hercules, CA). Each well was queried for fluorescence to determine the quantity of positive events (droplets), and the results were displayed as dot plots (Fig. 1).

### Quantitative PCR (qPCR)

For qPCR, the amplification of HTLV-1 *tax* and *beta-actin* were singleplexed in a 96-well plate. The final volume of each reaction was 25 μl (10 μl of DNA at a concentration of 10 ng/μl, 12.5 μl of 2× Universal TaqMan Mastermix (Applied Biosystems, Foster City, CA) and 2.5 μl of the primers/probe). Amplification and data acquisition were carried out in a Viia7 thermocycler (Applied Biosystems, Foster City, CA). Thermal cycling conditions were as follows: hold at 50 °C for 2 min, a second hold at 95 °C for 10 min, 45 cycles of 95 °C for 15 s and 60 °C for 1 min, followed by a final hold at 25 °C for 2 min.

### Data analysis and proviral load calculation

For ddPCR analysis, QuantaSoft software version 1.3.2.0 (Bio-Rad, Hercules, CA) was used to quantify the copies/μl of each queried target per well. Thresholds were determined manually for each experiment, according to the negative controls, which included a no template control and DNA from a healthy volunteer. Droplet positivity was determined by fluorescence intensity; only droplets above a minimum amplitude threshold were counted as positive. Negative controls were included in each experiment and resulted in less than two positive droplets. All samples were run in duplicate, unless otherwise specified, and the PVL is the average of the two measurements.

For qPCR analysis, Viia7 software version 1.2.1 (Applied Biosystems, Foster City, CA) was used to quantify HTLV-1 *tax* and *beta-actin*. All samples were run in triplicate and the average quantity was used to calculate the normalized PVL.

For both assays, the PVL was calculated as the percentage of infected cells using the following formula (Nagai et al. 2001a):

$$ \mathrm{PVL}=\left(\frac{\mathrm{quantity}\;\mathrm{of}\;\mathrm{HTVL}-1\;\mathrm{tax}}{\left(\frac{\mathrm{quantity}\;\mathrm{of}\;\mathrm{housekeeping}\;\mathrm{gene}}{2}\right)}\right)\times 100 $$

### Statistical methods-PVL normalization

Data normalization was accomplished by applying the log (base 2) transformation, calculating the mean and standard deviation (SD), and defining the lower (mean-2SD) and upper (mean + 2SD) values of the expected range for each assay type. These values were used to convert the log transformed values to a percentage of the expected range for each assay type, by subtracting the lower range value for the assay first then dividing by the difference of the upper and lower values of the expected range for the assay. Linear relationship of the post-normalized values was assessed using Pearson correlation. The “pROC” function supported in the statistical programming language R (http://cran.us.r-project.org/) was used per ROC analysis.
